# Acute pulmonary embolism as a complication in a young male patient with a left popliteal venous aneurysm

**DOI:** 10.1590/1677-5449.202200732

**Published:** 2023-07-17

**Authors:** Emmanuel Contreras-Jiménez, Jose I. Martínez-Quesada, Montserrat W. Miranda-Ramirez, Javier E. Anaya-Ayala, Luis H. Arzola-Flores, Santiago Mier y Terán-Ellis, Cesar Cuen-Ojeda, Carlos A. Hinojosa

**Affiliations:** 1 Instituto Nacional de Ciencias Médicas y Nutrición Salvador Zubirán, Dirección de Cirugía, Sección de Angiología, Cirugía Vascular y Endovascular, Ciudad de México, México

**Keywords:** popliteal vein, aneurysm, pulmonary embolism, veia poplítea, aneurisma, embolia pulmonar

## Abstract

Venous aneurysms are rare and have a prevalence of 0.1 to 0.2% in the reported series. Typically, patients do not present any symptoms, but are prone to develop deep venous thrombosis (DVT) and the most feared complication, pulmonary embolism (PE). We present the case of a previously healthy 36-year-old man who presented at the emergency department with tachycardia, dyspnea, and pleuritic pain. A thoracic computed tomography angiography (CTA) confirmed the diagnosis of acute pulmonary embolism. He was treated with systemic thrombolysis and anticoagulation. In the further workup of the cause of the embolism, computed tomography revealed a fusiform dilation of the left popliteal vein measuring 3 by 3 centimeters (cm) with an incomplete filling defect because of thrombus presence. The patient underwent open surgical repair. At one month follow-up, he was asymptomatic, and an ultrasound revealed complete patency of the popliteal vein without dilatation or thrombus.

## Resumo

Aneurismas venosos são raros, tendo uma prevalência de 0,1 a 0,2% nas séries relatadas. Os pacientes não costumam manifestar sintomas; entretanto, são propensos a desenvolver trombose venosa profunda e a complicação mais temida, embolia pulmonar. Apresentamos o caso de um homem de 36 anos previamente hígido que chegou ao serviço de emergência queixando-se de taquicardia, dispneia e dor pleurítica. Uma angiotomografia computadorizada confirmou o diagnóstico de embolia pulmonar aguda. O paciente foi tratado com trombólise sistêmica e anticoagulantes. Em exames posteriores para investigar a causa da embolia, uma tomografia computadorizada revelou dilatação fusiforme da veia poplítea esquerda medindo 3x3 cm, com enchimento incompleto devido à presença de um trombo. O paciente foi submetido a reparo cirúrgico aberto. No seguimento de 1 mês, ele estava assintomático, e uma ultrassonografia revelou a patência completa da veia poplítea, sem dilatação ou trombo.

## INTRODUCTION

Vein aneurysms are rare lesions and have a prevalence of 0.1 to 0.2% and can occur in any vein of the body.^1^ In 1926, Stevenson published what seems to be the first report of a pseudoaneurysm in the popliteal artery and vein.^2^ However, May and coauthors reported the first well-documented case in 1968.^3^ Most of these cases are asymptomatic, but diagnosis is nevertheless important since they can have catastrophic complications such as massive pulmonary embolism (PE) secondary to aneurysm thrombosis. The patient described in this case report gave consent to publication and the actual manuscript was approved by our institutional ethics committee (SCI-4110-22-22-1).

## CASE REPORT

**Figure 1:**
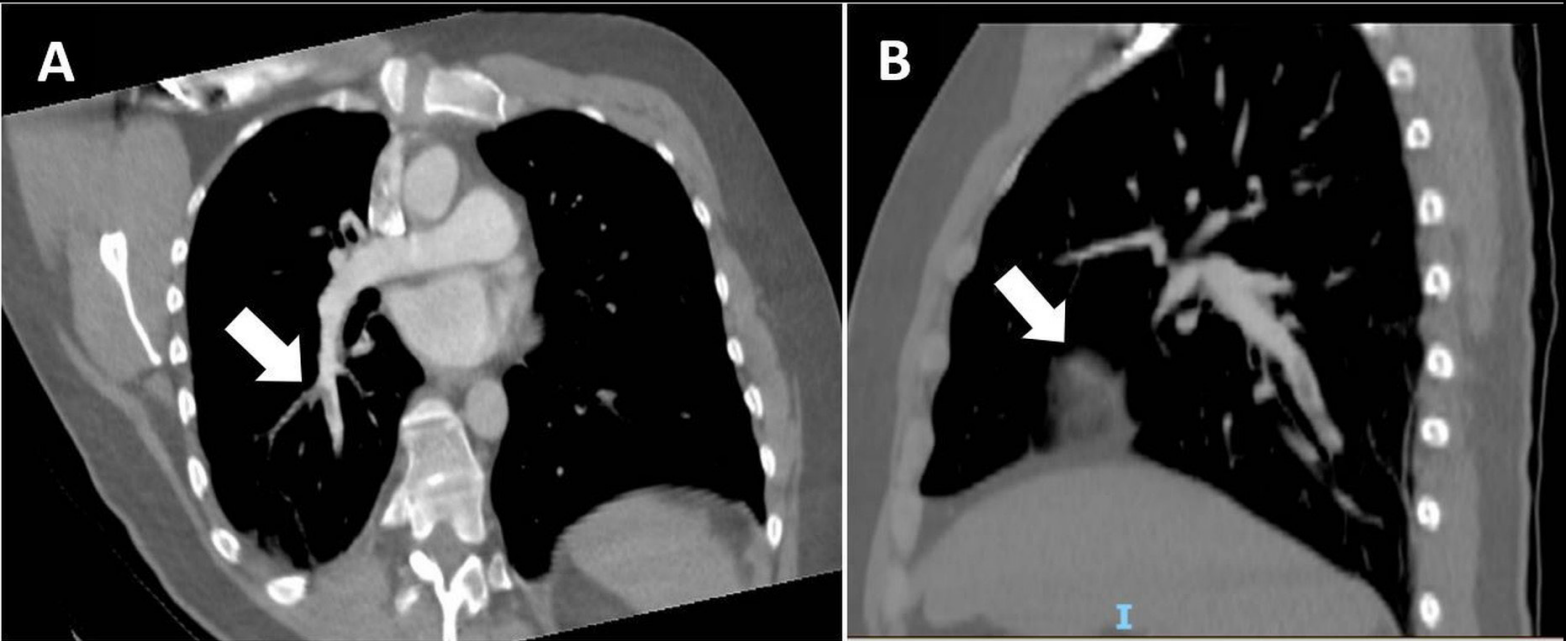
Thoracic computed tomography angiography (A) axial and (B) sagittal demonstrating pulmonary embolism (arrow).

**Figure 2:**
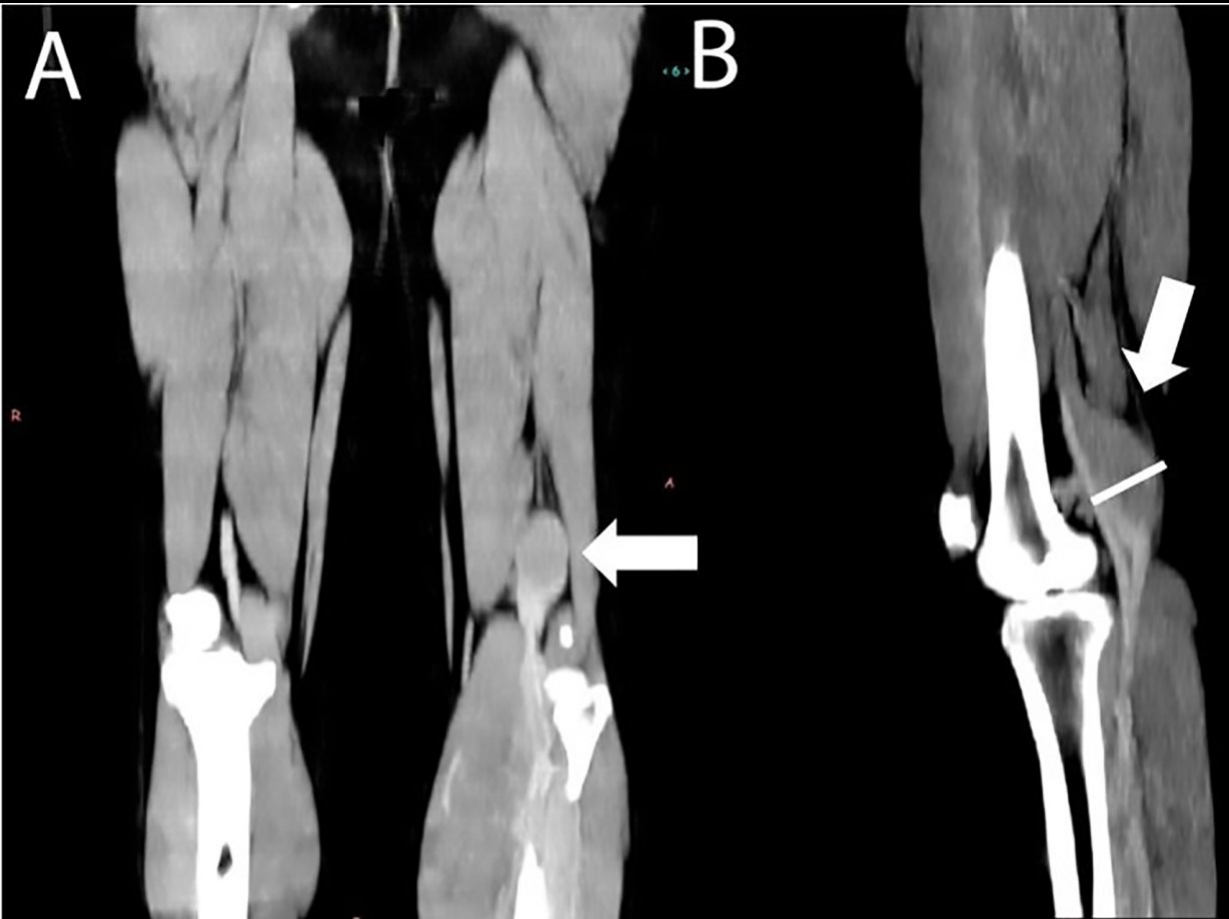
Lower limb computed tomography (a) coronal and (b) sagittal showing 3 x 3-centimeter dilatation of the popliteal vein (arrow).

We present the case of a previously healthy 36-year-old male patient who presented to the emergency department of an outside facility with sudden onset of tachycardia, dyspnea, hypoxemia, and thoracic pain. A thoracic computed tomography angiography (CTA) was performed (Figure 1) and the patient was diagnosed with acute PE and was immediately treated with systemic thrombolysis in an outside facility (40 milligrams of Tenecteplase) and anticoagulation (apixaban 5 milligrams BID) without complications or bleeding. During workup to rule out deep vein thrombosis (DVT), a fusiform dilation of the left popliteal vein measuring 3 by 3 centimeters (cm) and with an incomplete filling defect was detected on computed tomography of the lower limb, and the patient was referred to the vascular surgery service at our institution (Figure 2).

Physical examination revealed a palpable and non-painful mass on the popliteal fossa, both extremities without edema, and palpable pulses on both feet. The patient was unaware of this lesion. Anticoagulation with apixaban was continued and surgery was scheduled.

**Figure 3:**
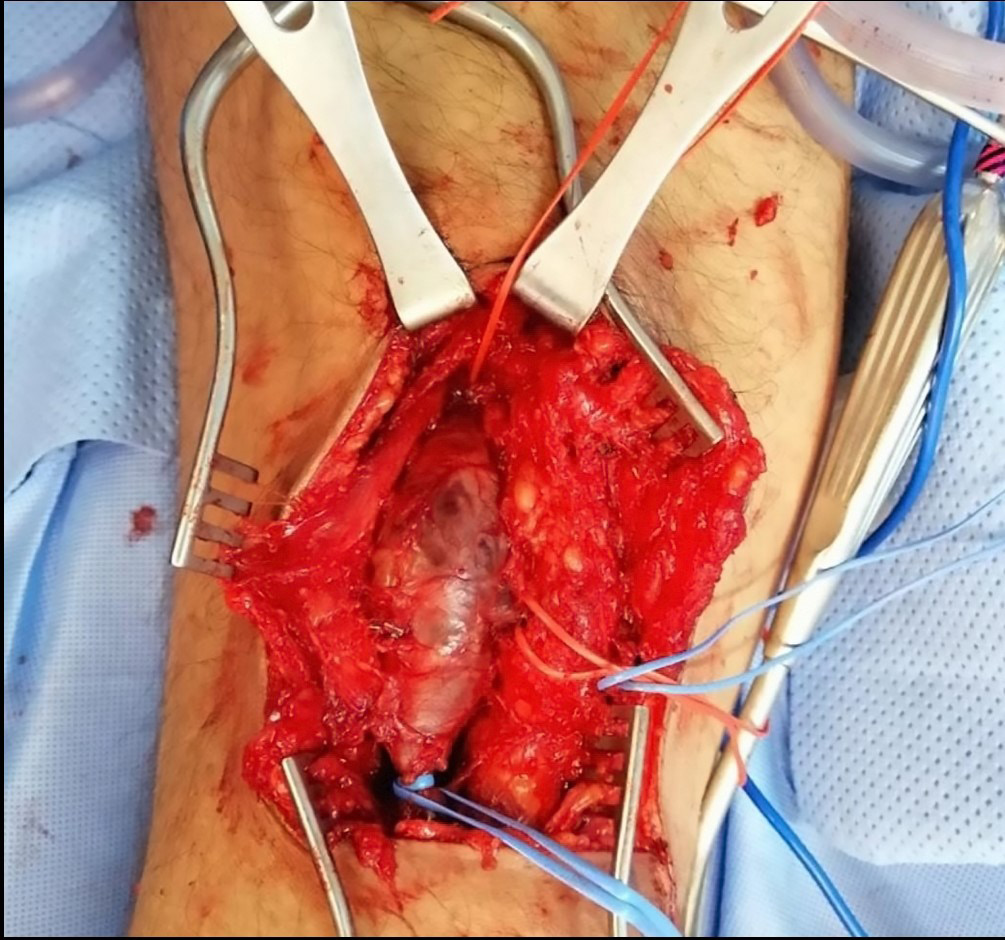
Posterior view of the popliteal fossa showing the popliteal vein aneurysm.

Surgery was performed under general anesthesia with the patient in a prone position, a posterior popliteal approach was used with a lazy S incision, and the popliteal vein was dissected and controlled with vascular loops (Figure 3). Afterward, 4000 IU of unfractionated heparin was administered (75 IU per kilogram) and two Satinskys were used to clamp and exclude the aneurysm. Subsequently, venotomy and thrombectomy were performed with excess vein wall resection and there was no residual thrombus inside the vein. A double-armed 5-0 polypropylene suture was used for aneurysmorrhaphy of the popliteal vein. Finally, patency was confirmed with an audible Doppler biphasic signal. The patient was tested for hypercoagulable states and thrombophilias but had no explained or provoked source of thrombi or embolism. The presence of the thrombus was attributed to the aneurysmal formation.

**Figure 4:**
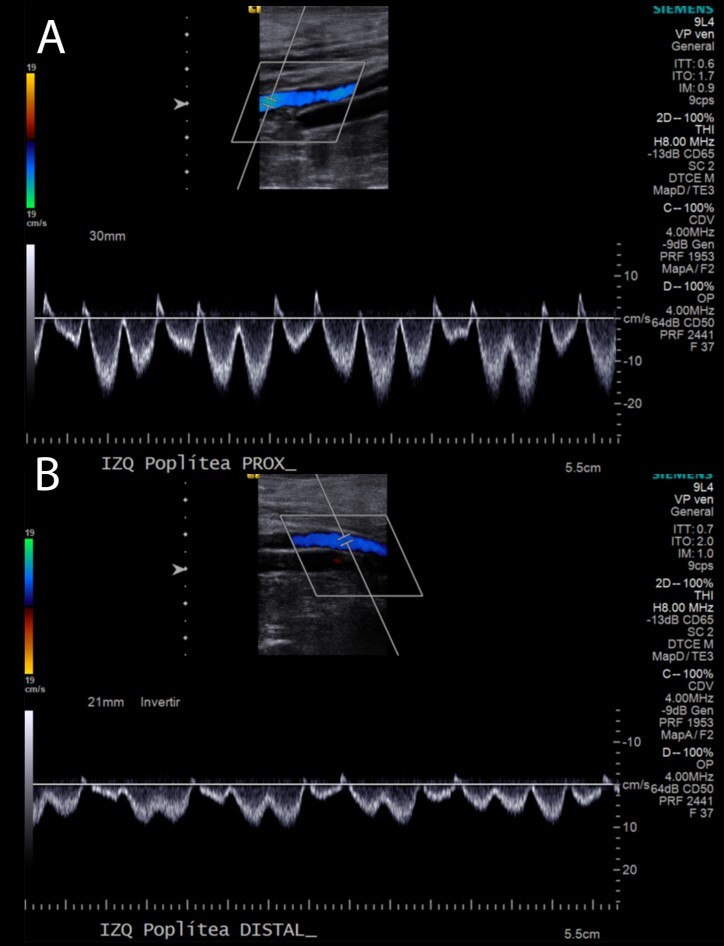
One-month follow-up popliteal duplex ultrasound evaluation in color doppler demonstrating patency (A) of the surgically reconstructed popliteal vein without aneurysmal dilation or presence of thrombus (B).

The patient was discharged 48 hours later, with no complications related to the procedure or during the postoperative period. On the follow-up visit at 1 year, the patient was asymptomatic, and an ultrasound found complete patency of the popliteal vein (Figure 4).

## DISCUSSION

There are currently no guidelines about this disease because of the low incidence of venous aneurysms and the consequent lack of information reported in the literature. The pathogenesis of these rare lesions is unknown.^4-5^

A systematic review in 2006, reported a total of 105 case reports of popliteal vein aneurysms.^6^ Their causes have been associated with congenital abnormalities, trauma, chronic inflammation, surgical manipulation, and arteriovenous fistula. Also, one case was associated with an osteoma.^7^

The first case of recurrent PE was documented by Dahl et al.^8^ Some cases tend to be asymptomatic and the aneurysms are accidentally identified due to other causes.^9-12^ Nevertheless, when there are associated symptoms, these are usually of pulmonary origin and chronic venous disease.^13^ In most cases, PE is present and is the first manifestation of a venous aneurysm.^14-18^ Although rare, a patient with a lower extremity aneurysm can debut with DVT.^19-20^ When the patient arrived at our institution, he had a palpable mass in the popliteal fossa, but he wasn’t aware of it and denied any other symptoms. Local symptoms like a palpable mass, erythema, symptoms due to compression of adjacent structures, and pain can present less frequently.^21^

It is important to offer surgical treatment at the time of diagnosis due to the severity of the complications. Nevertheless, there are reports of conservative management without complications of aneurysms less than 2 cm and no evidence of thrombus.^13^ In this case, the patient’s aneurysm measured 3 by 3 cm and debuted as a PE, due to this, the best approach was the repair of the aneurysm to prevent a second acute DVT event.

Currently, there are multiple approaches described for the management of this pathology, however, there are no current guidelines or indications that are considered standard, since all the current evidence is based on case reports or series describing treatment ranging from open management, with aneurysm resection and venorrhaphy, to endovascular management with stent placement and aneurysm exclusion. The surgical decision is made based on the symptoms and complications, the characteristics of the aneurysm, and the experience of the vascular surgeon. We recognize the limitations of the present case study, such as the fact that there was no formal indication for use of a thrombolytic agent according to the current international guidelines. However, the patient arrived at our institution with a prior history of thrombolytic therapy at an outside hospital. This case illustrates and reinforces the importance of adherence to the current clinical practice PE guidelines for all clinicians.^22^ Thrombolytic therapy should be recommended only for high-risk PE (hemodynamic instability + clinical parameters of PE severity + right ventricle [RV] dysfunction + elevated cardiac troponin levels) and for Intermediate High Risk (clinical parameters of PE severity + RV dysfunction + elevated cardiac troponin levels). Also, no histological analysis of the resected popliteal vein was conducted.

In conclusion, venous aneurysms are infrequent. It is important to make a prompt diagnosis with further management to prevent potentially lethal complications or suspect a venous aneurysm as a possible differential diagnosis of PE with no apparent cause.
